# Integrative taxonomy reveals a new species of the soapfish genus *Rypticus* (Teleostei: Grammistidae) from the eastern Atlantic Ocean

**DOI:** 10.1111/jfb.70132

**Published:** 2025-07-21

**Authors:** Gabriel Soares Araujo, Cláudio L. S. Sampaio, Luiz A. Rocha, Carlos E. L. Ferreira

**Affiliations:** ^1^ Center for Marine Biology University of São Paulo São Sebastião Brazil; ^2^ Laboratório de Ictiologia e Conservação Universidade Federal de Alagoas Penedo Brazil; ^3^ Department of Ichthyology California Academy of Sciences San Francisco California USA; ^4^ Departamento de Biologia Marinha Universidade Federal Fluminense Niterói Brazil

**Keywords:** Biogeography, cryptic species, DNA barcode, Gulf of Guinea, Isthmus of Panama, reef fish

## Abstract

A new species of the soapfish genus *Rypticus* is described based on 14 specimens from the eastern Atlantic Ocean. The new species was previously misidentified as the greater soapfish, *R. saponaceus*, due to their similar appearance. However, it differs from *R. saponaceus* in several key characteristics, including a comparatively shorter head, snout and upper jaw, and a deeper body. Molecular data, obtained from the mitochondrial cytochrome oxidase I gene, strongly suggest the monophyly of the new species and support its description as new.

## 1 INTRODUCTION

The fishes of the family Grammistidae are small to medium‐sized cryptic inhabitants of reefs and rocky shores in tropical and temperate shallow waters around the world. Members of this group show a diversity of habitat preferences, with some preferring muddy bottoms and low water clarity, and others restricted to reef environments with clear waters (Bohlke & Chaplin, 1996). The family is characterized by having the entire upper edge of the opercle bound to the skull by a membrane, less than 10 procurrent caudal‐fin rays, preopercle with one to three spines, and in having the innermost ventral ray attached to the belly by a membrane (Baldwin & Johnson, 1993; Heemstra et al., 2002). The genera *Grammistes* Bloch & Schneider 1801, *Grammistops* Schultz 1953, *Pogonoperca* Günther 1859, and *Rypticus* Cuvier 1829 secrete substantial amounts of toxic, soap‐like mucus as a stress response, earning them the common name soapfishes (Baldwin & Johnson, 1993; Randall et al., 1971).

*Rypticus* is the second most diverse genus in the family, with 10 valid species, three of which are in the eastern Pacific and seven in the Atlantic Ocean. Most Atlantic species are restricted to the western Atlantic, with only two also occurring in the eastern Atlantic, the greater soapfish *Rypticus saponaceus* (Bloch & Schneider, 1801) and the spotted soapfish *Rypticus subbifrenatus* Gill, 1861. *Rypticus saponaceus* is recorded in Bermuda, south Florida, Gulf of Mexico, throughout Caribbean to southern Brazil, in the western Atlantic, in the Mid‐Atlantic Ridge tropical islands and from Mauritania to Angola, including Cape Verde and São Tomé and Príncipe, in the eastern Atlantic (Fermon et al., 2022; Parenti & Randall, 2020). *Rypticus subbifrenatus* is recorded from southern Florida, the Caribbean Sea, to southern Brazil in the western Atlantic, and from Senegal to Angola, including the island of São Tomé and Príncipe, in the eastern Atlantic (Fermon et al., 2022; Parenti & Randall, 2020).

Carlin et al. (2003) found considerable genetic differences, possibly at species‐level, among individuals identified as *R. saponaceous* from the western and eastern Atlantic, suggesting that the african population may be a cryptic undescribed species. In this study, we describe a new species of *Rypticus* from the eastern Atlantic, integrating both morphological and molecular data. The new species resembles *R. saponaceus* but exhibits distinct morphological characteristics, setting it apart from all other congeners. We incorporated molecular data to test the monophyly of the new species, evaluated the genetic distances among its congeners and conducted lineage delimitation tests.

## 2 MATERIALS AND METHODS

The care and use of experimental animals complied with Republic Democratic of São Tomé and Príncipe animal welfare laws, guidelines and policies as approved by the Direcção das Pescas of the Democratic Republic of São Tomé and Príncipe.

Fourteen specimens of *Rypticus* sp. from Cape Verde, Ghana, São Tomé and Príncipe, and Togo were examined (Table S1). Morphometric and meristic data were taken following Guimaraes (1999). Measurements were obtained with callipers to 0.05 mm. Range of counts and measurements are presented first, followed by values for the holotype in parentheses. Counts of median fin rays and vertebrae were taken from digitally scanned radiograph images of the holotype and all paratypes. Description of colour is based on live type specimens as well as after preservation in ethanol. The type series was deposited in the Museu de Zoologia da Universidade Federal da Bahia (UFBA), and the Muséum National d'Histoire Naturelle (MNHN) ichthyological collections.

We obtained 103 sequences of the cytochrome oxidase I gene (COI) of *Rypticus* from GenBank and the Barcode of Life Data System (BOLD), including sequences of the new species from Cape Verde, Ghana and São Tomé and Príncipe (see acknowledgements). One of the paratypes (MNHN2002‐158) was sequenced (GenBank accession number JX093905) and therefore constitutes a genseq‐2 cytochrome oxidase I (Chakrabarty et al., 2013). Additionally, the sequences’ Genbank accession numbers, PV137739 and PV137741, which were exclusively employed in genetic analyses, are associated with voucher specimens deposited in the ichthyological collection of the Smithsonian Institution's National Museum of Natural History (USNM405085 and USNM405086, respectively). Consequently, both sequences constitute a genseq‐3 cytochrome oxidase I. GenBank and BOLD accession numbers for each sequence are available in Table S1. The sequences were aligned in the software MEGA 7.0 (Kumar et al., 2015), using the Clustal W algorithm (Thompson et al., 1994). The genetic distance was estimated using MEGA 7.0, through the Kimura 2‐parameter distance model (K2P) (Kimura, 1980). The nucleotide substitution model suggested by the jModelTest 2.1 (Darriba et al., 2012) was HKY+G.

We conducted two phylogenetic Bayesian analyses. The first one was executed in MrBayes 3.2.6 (Ronquist et al., 2012), with two independent runs of four concomitant Markov chain Monte Carlo (MCMC) for 40 million generations and sampling parameters every 4000 generations. The second method was implemented in the software BEAST 2.6.2 (Bouckaert et al., 2019) assuming a strict clock with the clock.rate parameter set to 0.0075 and the Yule model. Two independent analyses were performed, each with a chain length of 80 million generations. Trees and parameters were sampled every 8000 generations and the first 20% of the samples were discarded as burn‐in. The software Tracer 1.5 (Rambaut et al., 2018) was used to check the results of the runs, and the analyses were combined with LogCombiner 2.6.2 (Bouckaert et al., 2019). A maximum clade credibility tree was obtained through TreeAnnotator 2.6.2 (Bouckaert et al., 2019). The outgroup in both analyses was *Grammistes sexlineatus* (Thunberg 1792) due to its close phylogenetic relationship with *Rypticus* (Craig & Hastings, 2007).

Finally, we performed three lineage delimitation tests: the Multi‐rate Poisson Tree Processes (mPTP; Kapli et al., 2017), the Generalized Mixed Yule Coalescent (GMYC; Fujisawa & Barraclough, 2013) and the Assemble Species by Automatic Partitioning (ASAP; Puillandre et al., 2021).

## 3 RESULTS

**FIGURE 1:**
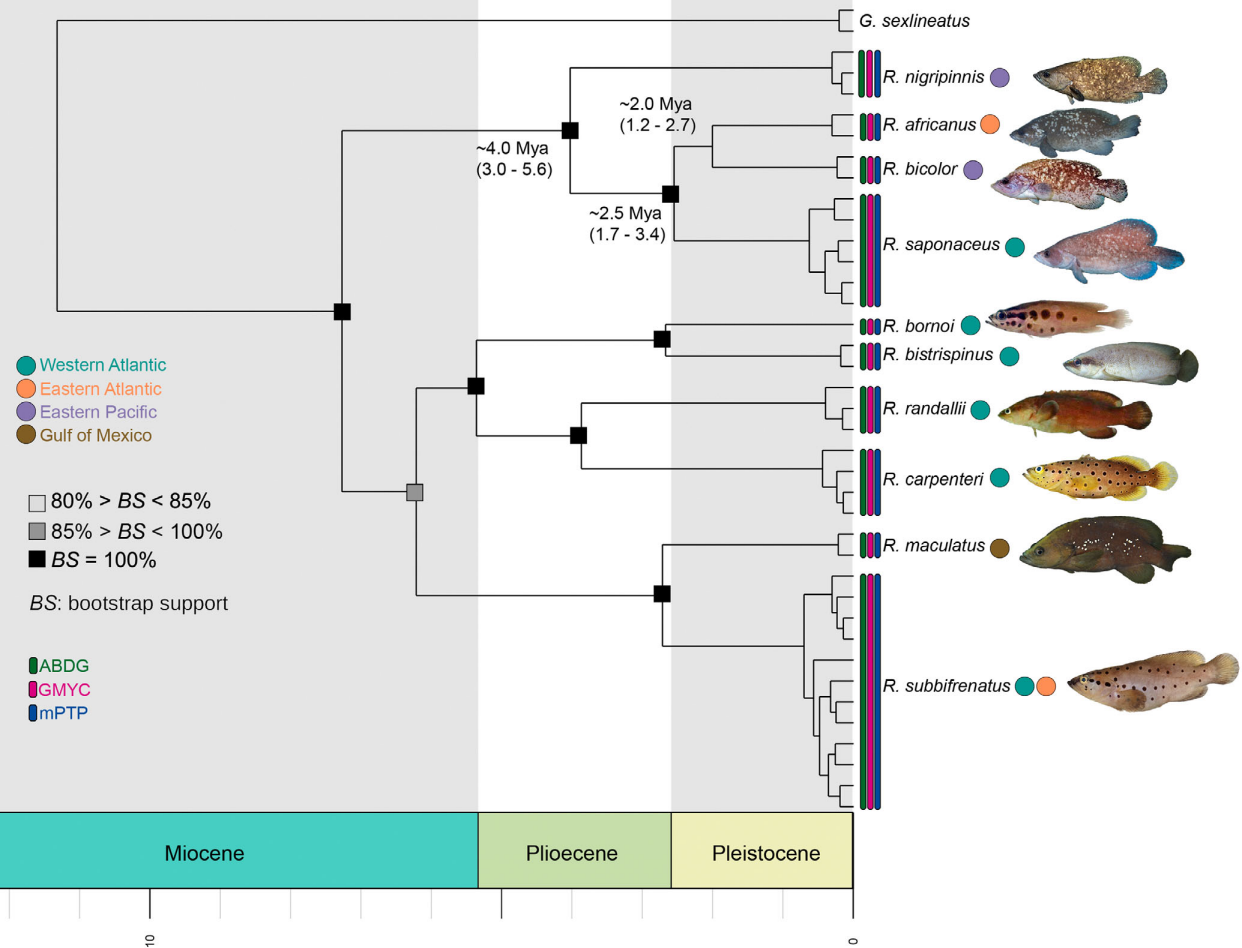
Time‐calibrated phylogeny of the genus *Rypticus* based on mitochondrial COI gene sequences. Values near the nodes represent estimated divergence times (Mya). The 95% highest posterior density intervals are shown in parentheses. Coloured circles represent the biogeographic regions where species of *Rypticus* are distributed. Vertical bars depict the results of lineage delimitation tests. Photo credit: Alisson and Carlos Estapé, Carole C. Baldwin, Frank Krasovec, Robert Robins, Zachary Randall and the Shorefishes of the Greater Caribbean online information system (https://biogeodb.stri.si.edu/caribbean/en/pages).

Both phylogenetic analyses recovered highly similar topologies, delineating two primary clades separated by an estimated divergence time of 7.2 million years according to the BEAST results (Figure 1). One clade comprised five western Atlantic species and the amphi‐Atlantic *R. subbifrenatus*. The other clade encompassed *R. saponaceus* along with eastern Pacific *Rypticus* species, namely, *Rypticus nigripinnis* Gill 1861 and *Rypticus bicolor* Valenciennes 1846. Inter‐specific genetic distances ranged from 2.4% to 11.2%, while the maximum intra‐specific distance was only 0.7% (Table 1). These results support the monophyly of all valid species of *Rypticus* with maximum support values, except for *R. saponaceus*.

**TABLE 1:** K2P genetic distance values for *Rypticus* species.

	*R. africanus*	*R. bicolor*	*R. bistrispinus*	*R. bornoi*	*R. carpenteri*	*R. maculatus*	*R. randalli*	*R. saponaceus*	*R. subbifrenatus*
*R. africanus*	**0.1%**								
*R. bicolor*	2.8%	**0.0%**							
*R. bistrispinus*	11.2%	8.7%	**0.1%**						
*R. bornoi*	10.2%	9.3%	5.0%	**0.0%**					
*R. carpenteri*	8.1%	7.1%	8.3%	8.3%	**0.1%**				
*R. maculatus*	9.0%	7.2%	9.5%	9.6%	8.2%	**0.2%**			
*R. randalli*	9.5%	8.7%	9.1%	9.5%	5.9%	8.7%	**0.1%**		
*R. saponaceus*	3.3%	2.4%	10.3%	10.5%	7.7%	8.4%	9.0%	**0.3%**	
*R. subbifrenatus*	8.6%	7.6%	10.4%	11.0%	8.0%	3.7%	8.6%	8.2%	**0.7%**

*Note*: Intraspecific genetic distance values are shown in bold.

Phylogenetic analyses recovered two well‐supported monophyletic groups within sequences identified as *R. saponaceus*, one from the western Atlantic and another from the eastern Atlantic (Figure 1). This topology mirrored that of Carlin et al. (2003) based on the cytochrome B gene (Cytb), where western Atlantic samples (Grenada, Barbados and Brazil) and eastern Atlantic samples (Cape Verde and São Tomé and Príncipe) formed distinct clades. Given that the type localities of *R. saponaceus* (Florida, United States; Courtenay, 1967) and its synonyms, *Rypticus microps* Castelnau (ex Broussonet) 1955 (Bahia, Brazil) and *Eleutheractis coriaceus* Cope 1871 (St. Martin Island, West Indies), are exclusively western Atlantic and that the western Atlantic samples used here form a monophyletic group (as in Carlin et al., 2003), we restrict the name *R. saponaceus* to this clade and describe the eastern Atlantic clade as *Rypticus africanus* n. sp. (see Systematics).

The relationships between these clades remained unresolved due to changing topology and moderate support values. The BEAST analysis recovered the topology *R. saponaceus* (*R*. *africanus* + *R. bicolor*), whereas the MrBayes analysis suggested *R. bicolor* (*R*. *africanus* + *R. saponaceus*). The COI‐based time tree estimated a divergence time of approximately 2.5 million years between *R*. *africanus* and *R. saponaceus*. The genetic divergence between these two groups was substantial (3.3%; Table 1), highlighting a notable genetic distinction between individuals from these regions. Conversely, intra‐lineage genetic distances were minimal, ranging from 0.0% to 0.3%, indicating a high degree of genetic similarity within populations (Table 1). All three lineage delimitation tests were congruent with the tree topologies, consistently identifying 10 well‐defined lineages. These lineages correspond to the eight currently valid species of the genus included here and the two distinct lineages previously identified as *R. saponaceus* (Figure 1).

The combined indication from phylogenetic analyses, lineage delimitation tests, the observed genetic divergence, the biogeographic compartmentalization and the morphological differences (see Systematics) between western and eastern Atlantic individuals identified as *R. saponaceus* strongly support the existence of a previously unrecognized *Rypticus* species along the west African coast and islands.

## 4 SYSTEMATICS

*Rypticus africanus* Araujo, Sampaio, Rocha & Ferreira, new species.

**FIGURE 2:**
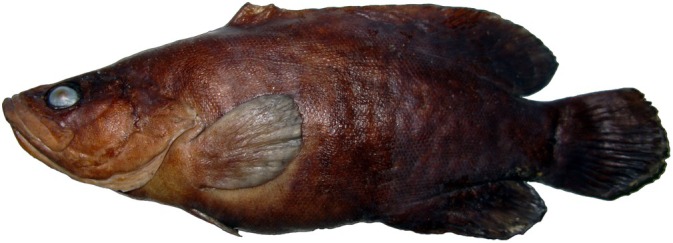
Holotype of *Rypticus africanus*. UFBA 2803, 142 mm SL from São Tomé and Príncipe, Gulf of Guinea.

**Zoobank registration:**

urn:lsid:zoobank.org:act:ABFC5A77‐DB51‐4901‐ABF3‐64456A4E6A36.

(Figures 2 and 4; Tables 1, 2).

**TABLE 2:** Counts and measurements (of type specimens) of *Rypticus africanus*.

	UFBA 2803^a^	UFBA 2802	UFBA 2802	UFBA 2802	MNHN 2002–0158
Standard length (mm)	142.0	146.0	149.4	156.4	173.1
Head length	34.9	35.0	35.8	35.8	34.4
Snout length	5.6	6.1	6.3	5.8	5.8
Upper jaw length	14.0	15.0	14.7	14.5	15.0
Eye diameter	6.0	6.2	5.6	5.7	7.5
Interorbital width	3.3	3.0	3.4	3.1	3.7
Predorsal distance	39.7	39.7	40.4	39.6	40.5
Body depth	38.2	36.3	37.0	37.7	36.1
Dorsal‐fin base	54.5	51.5	50.4	49.7	48.6
Total vertebrae	24 (10 + 14)	24 (10 + 14)	24 (10 + 14)	24 (10 + 14)	24 (10 + 14)
Anal‐fin rays	16	16	16	16	16
Dorsal‐fin rays	III‐24	III‐24	III‐22	III‐24	III‐24
Pectoral‐fin rays	16	17	18	17	16
Caudal‐fin rays	25	25	25	25	25

*Note*: Measurements other than standard length are shown as percentages of the standard length.

^a^ Holotype.

**English proposed common name:**

African soapfish.

**Portuguese (São Tome and Príncipe) proposed common name:**

Peixe sabão africano.

**Synonymy (in part):** *Rypticus saponaceus*: Cuvier and Valenciennes (1829); Poll (1949); Cadenat (1950); Poll (1954); Cadenat (1960); Debelius (1997); Afonso et al. (1999); Guimarães (1999); Kuiter (2004), Wirtz et al. (2007); Wirtz et al. (2013); Wirtz et al. (2014); Heemstra and Anderson (2016); Reiner (2019); Brown et al. (2019); Almeida and Alves (2019); Parenti and Randall (2020); Fermon et al. (2022).

**FIGURE 3:**
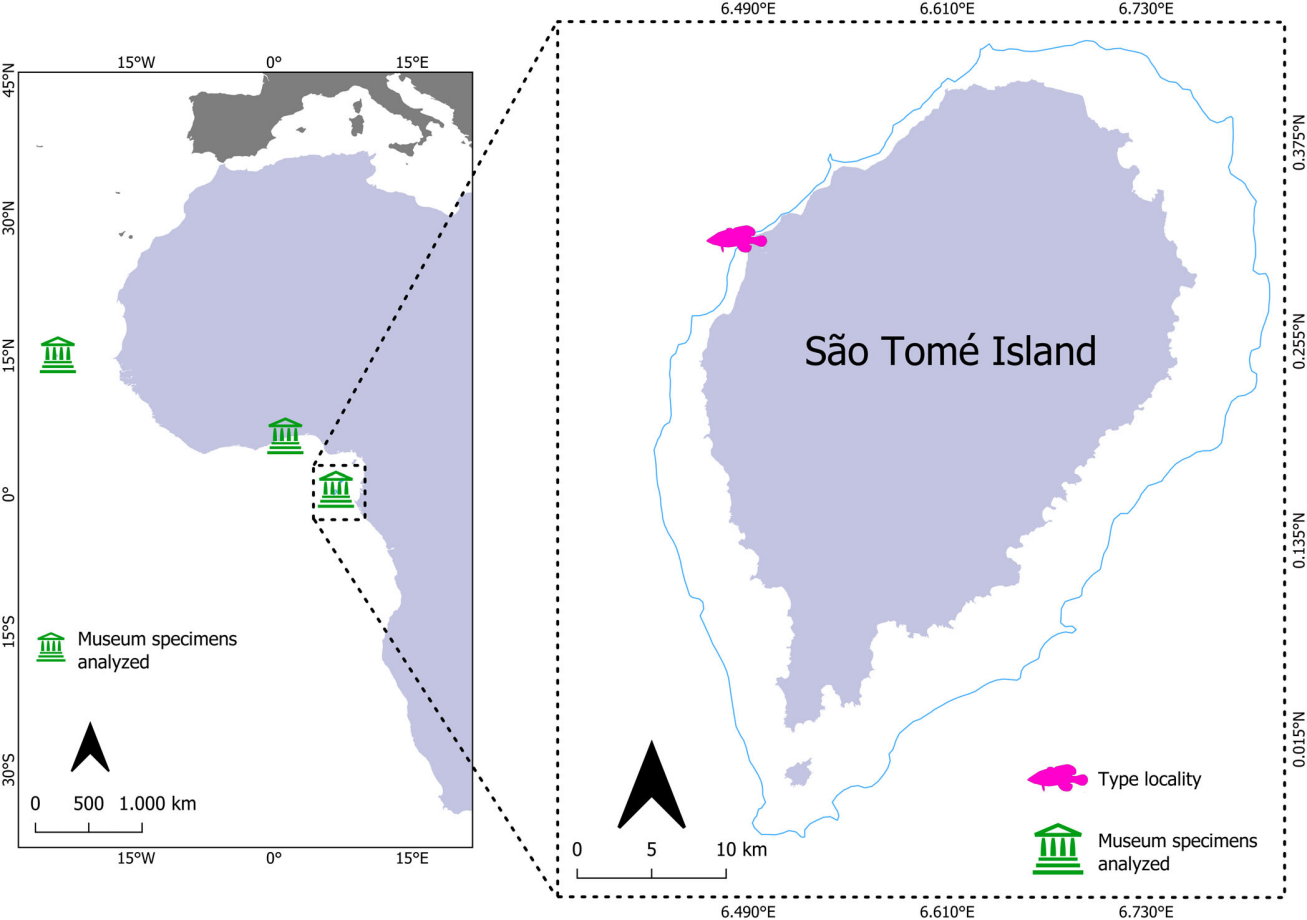
Collection sites of *R. africanus* samples analysed morphologically. The 200 m isobath is highlighted in blue.

**Holotype:** UFBA 2803, 142 mm standard length (SL) [Figure 2], Diogo Vaz beach, north coast of São Tomé Island, São Tomé and Príncipe, 17 m depth, 0°19′ N, 6°29′ E, collected by C.L.S. Sampaio, C.E.L. Ferreira, S.R. Floeter, J.L. Gasparini, L.A. Rocha, and P. Wirtz, 13 February 2006 (Figure 3).

**Paratypes:** UFBA 2802 (3: 146.0–156.4 mm SL), collected with the holotype; MNHN 2002–0158, 173.1 mm SL, specimen purchased at a market in Ghana by Y. Fermon, 2002 (Figure 3).

**Non‐type specimens:** MNHN‐IC‐1887‐0359, 113 mm SL, São Vicente Island, Cape Verde, 16°54′N 25°0′W; MNHN‐IC‐1887‐0360, 114 mm SL, same data as MNHN‐IC‐1887‐0359; MNHN‐IC‐0000‐7840, 213 mm SL, São Tiago Island, Cape Verde, 14°55′N, 23°31′ W; MNHN‐IC‐1971‐0094 (2: 167–185 mm SL), Gulf of Guinea, 0°00′ N, 5°0′E; MNHN‐IC‐1962‐0083, 167 mm SL, São Tiago Island, Cape Verde, 15°7′ N, 23°24′ W; MNHN‐IC‐1941‐0027 (2: 160–187 mm SL), Porto Ingles, Cape Verde, 15°7′N, 23°15′W; MNHN‐IC‐1967‐0815, 150 mm SL, Togo, 6°10′N, 28°12′E (Figure 3).

### 4.1 Diagnosis

A species of *Rypticus* distinguished from its congeners by the following unique combination of characters: dorsal‐fin spines always three vs. two/typically two in *Rypticus bistrispinus* (Mitchill 1818), *Rypticus bornoi* Beebe & Tee‐Van 1928, *Rypticus courtenayi* McCarthy 1979, *Rypticus maculatus* Holbrook 1855, and *R. nigripinnis*, and almost always four in *Rypticus carpenteri* Baldwin & Weigt, 2012; head length 30.0%–35.3% of SL (average 33.5%) vs. 35.0%–39% (average 36.88%) in *R. bicolor*; body depth 34.2%–40.5% of SL (average 36.2%) vs. 26%–34% (average 30.0%) in *Rypticus randalli* Courtenay, 1967; body brown to dark grey, with the head and sides displaying numerous or sparse pale, spots of variable size vs. lighter background coloration with several widely scattered small dark spots; head shorter than body depth vs. head larger than body depth in *R. saponaceus*.

### 4.2 Description

Based on 14 specimens, 112–215 mm SL. Counts and morphometric data of the types are summarized in Table 2. Values referring to the holotype in parentheses. Dorsal‐fin spines three, dorsal fin‐rays 22–24 (III‐24); total dorsal‐fin elements 25–27; anal‐fin elements 15–17 (16), modally 16; pectoral fin rounded with 16–18 rays (16), modally 16; total caudal‐fin rays 24–25 (25), modally 25. Head distinctly pointed, the dorsal profile nearly straight, its mean length 33.5% of SL (34.9%). Snout length 5.1–7.4% of SL (5.6%), mean = 6.2%; eye diameter 5.3–7.3% SL (6.0%), mean = 6.3%. Interorbital space slightly convex. Lower jaw extending anteriorly beyond upper jaw. Vertebrae 24 (10 + 14). Caudal fin rounded; origin of dorsal fin slightly posterior to upper end of gill opening; dorsal spines strong and membrane not incised; distal tip of pectoral fins and pelvic‐fin tips not reaching anus. Body moderately deep, mean depth at pectoral‐fin base 36.2% of SL (36.9%).

### 4.3 Colour in life

**FIGURE 4:**
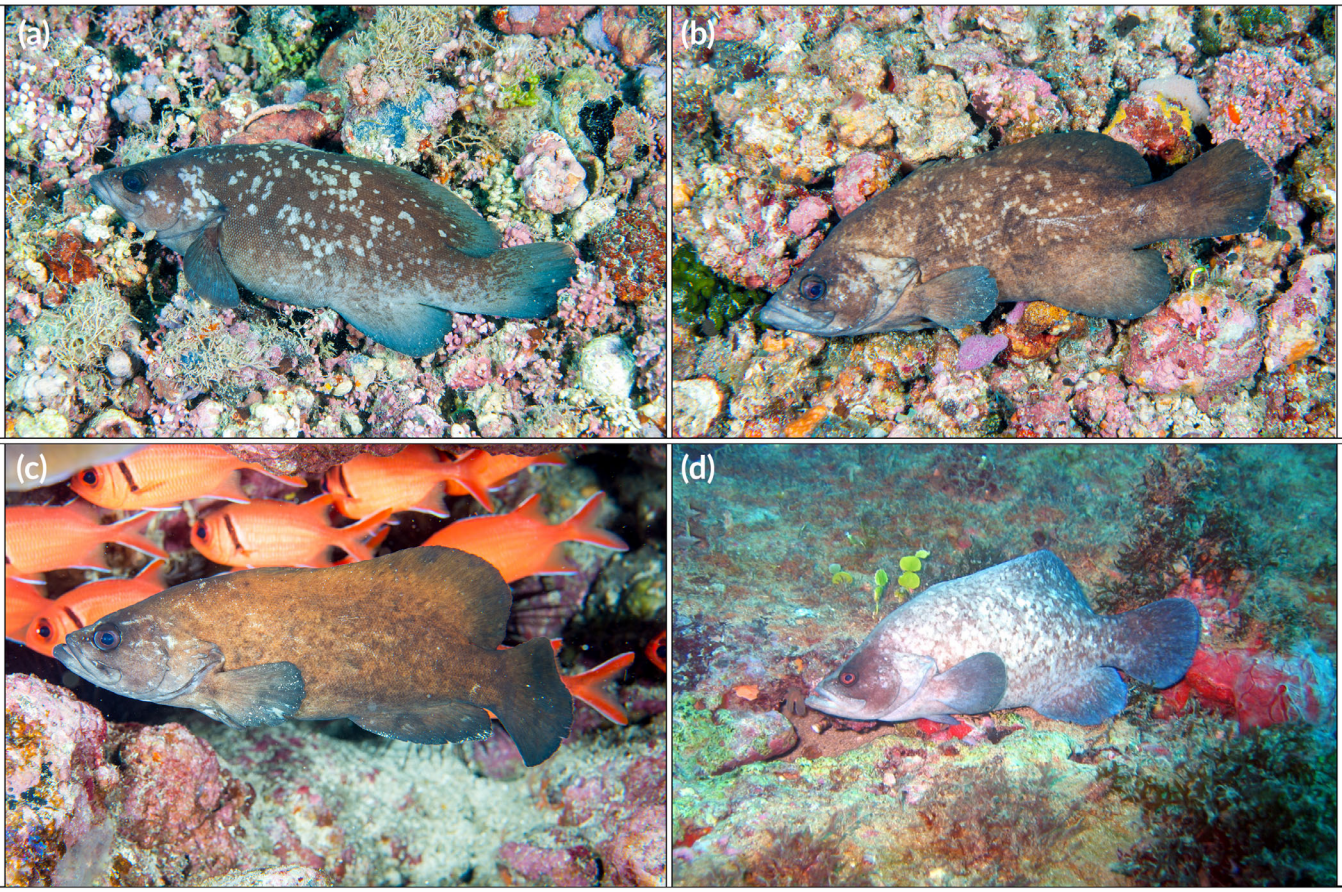
(a–c) *Rypticus africanus* from São Tomé and Príncipe, illustrating the variation in colour pattern between individuals: (a) São Tomé Island, (b, c) Príncipe Island and (d) *R. saponaceus* from Salvador, Bahia, Brazil, shown for comparison.

Specimens of *R. africanus* exhibit a brown to dark‐grey body colour, with the head and sides displaying numerous or sparse pale, round spots of variable size (Figure 4a–c). The fins are either the same colour as the body or lighter due to the concentration of pale spots. Smaller juveniles may display a predominantly bluish hue, pale lines radiating posteriorly from the eye and a distinct white stripe extending from the snout, between the eyes, and fading into the dorsal fin (see Debelius, 1997; Kuiter, 2004).

### 4.4 Colour in alcohol

Overall brownish body coloration, pectoral fins pale. Darker in the dorsal region and pale in ventral, no marks or distinctive spots on body (Figure 2).

### 4.5 Distribution

Based on examined specimens, DNA sequence data and previously reported geographic distribution of *R. saponaceus*, *R. africanus* likely occurs from Mauritania to Angola, and the oceanic Islands of Cabo Verde and the Gulf of Guinea, although we have not examined specimens throughout this range.

### 4.6 Habitat notes

The primary habitat of *R. africanus* is rocky reefs and rhodolith beds, in shallow clear waters to depths of 30 m. Reef habitats where the species was observed were mainly dominated by coralline algae (Figure 4a), encrusting sponges, sea fans, corals and gorgonians. Although it is not generally abundant, it may be common in some reef areas, especially in caves at 10–20 m depth, where it is usually found alone.

### 4.7 Etymology

The specific name *africanus* is given for the known distribution of the new species and its probable widespread distribution off the coast of west Africa.

### 4.8 Comparisons between *R. africanus* and *R. saponaceus*

Counts and measurements agree for the most part with those of *R. saponaceus*. The most conspicuous differences between these two species are in the lengths of head, snout, predorsal distance, and upper jaw and body depth. *Rypticus africanus* presents on average a shorter head length in SL (30%–35.3%, average 33.5% vs. 32–39%, average 36.1% in *R. saponaceus*), a shorter snout length in SL (5.1%–7.4%, average 6.2% vs. 6.0–9.0%, average 7.3%, in *R. saponaceus*), a shorter upper jaw length in SL (11.1%–16.0%, average 13.4% vs. 13.0%–17.0%, average 15.1%, in *R. saponaceus*), a shorter predorsal distance in SL (34.4%–40.7%, average 39.6% vs. 34.0%–46.0%, average 41.7%, in *R. saponaceus*), and a deeper body in SL (34.0%–40.5%, average 36.2%, vs. 31.0%–36.0%, average 33.0%, in *R. saponaceus*). Additionally, *R. africanus* typically exhibits a head shorter than the body depth vs. head longer than the body depth in *R. saponaceus*. Furthermore, in larger individuals, the average number of fully formed rakers is seven or eight in *R. africanus*, but never nine, vs. eight or nine in *R. saponaceus* (Courternay, 1967).

## 5 DISCUSSION

*Rypticus africanus* has historically been treated as *R. saponaceus* (Afonso et al., 1999; Debelius, 1997; Wirtz et al., 2013). Two taxonomic assessments based on the morphology and anatomy of *Rypticus* species failed to identify significant morphological distinctions among *Rypticus saponaceus* populations from the western and eastern Atlantic (Courtenay, 1967; Guimaraes, 1999). The challenge in species differentiation within the genus likely arises from widespread morphological conservatism, with species differentiation relying on combinations of characters (Courtenay, 1967; Guimaraes, 1999). Conversely, in a study utilizing the mitochondrial Cytb gene sequences from *Rypticus saponaceus* samples across the Atlantic, Carlin et al. (2003) identified significant genetic differentiation between *R. saponaceus* from the western and eastern Atlantic, suggesting the potential existence of at least one cryptic species. This investigation also highlighted the close phylogenetic relationship between *R. bicolor* and *R. saponaceus* (also proposed by Guimaraes, 1999) and estimated the divergence between these lineages at approximately 3.5 Mya and between western and eastern Atlantic *R. saponaceus* at approximately 3.3 Mya, emphasizing the role of the Isthmus of Panama in the diversification of this clade.

The topology and divergence time estimates provided by the analyses implemented here are similar to those found by Carlin et al. (2003). However, our comprehensive taxonomic sampling has enabled us to envisage a broader scenario for the diversification of *Rypticus*. Complementing the results found by Guimaraes (1999) and Carlin et al. (2003), we believe that the uplift of the Isthmus of Panama played a central and multifaceted role in the diversification of this *Rypticus* clade. Rather than a single and isolated vicariant event, our results indicate that the Isthmus of Panama acted as a dynamic and complex biogeographic barrier between the Atlantic and eastern Pacific faunas. Similar to evolutive scenarios proposed for other reef fishes (e.g. *Anisotremus* and *Sphoeroides*; Bernardi & Lape, 2005; Araujo et al., 2023), our analyses suggest two distinct separations mediated by the Isthmus of Panama, both occurring within a time frame very close to the complete closure of the Isthmus. The first separation, between *R. nigripinnis* and *R. saponaceus* (*R. africanus* + *R. bicolor*), is estimated to have occurred ~4 Mya, followed by a second separation between *R. saponaceus* and (*R. africanus* + *R. bicolor*), around 2.5 Mya.

In addition to closing the marine pathway between the eastern Pacific and the Atlantic, the rise of the Isthmus of Panama triggered changes in Atlantic Ocean currents, strengthening currents that carry western Atlantic waters towards the eastern Atlantic. These enhanced currents may have facilitated the dispersal of *R. africanus* ancestors to the eastern Atlantic. It is possible that this panorama of transoceanic dispersal was also facilitated by the Mid‐Atlantic tropical islands, which may have served as biogeographic stepping‐stones, given the presence of *Rypticus* aff. *saponaceus* in St. Helena and Ascension islands. Indeed, it has been proposed that *Rypticus* from the mid‐Atlantic islands may also represent a cryptic species, considering the reported morphological and genetic differences between specimens from this region and those from the western and eastern Atlantic (Brown et al., 2019; Carlin et al., 2003). However, a specimen of *R*. aff. *saponaceus* that we sequenced from Saint Helena exhibited negligible genetic divergence compared to samples from the western Atlantic. Lastly, it is worth noting that *R. subbifrenatus* is also recorded on both sides of the Atlantic and may also represent a species complex (Baldwin & Weigt, 2012).

## 6 CONSERVATION

Problem‐solving in diverse fields such as systematics, ecology, evolution and conservation policies relies on a thorough understanding of the basic unit of biodiversity, the species. One of the greatest challenges related to advancing biological knowledge globally is the delay between the discovery of a species and its formal description, estimated to be around 21 years (Fontaine et al., 2012; Pinheiro et al., 2019). Symbolically, 22 years have passed since the detection of a cryptic lineage identified as *R. saponaceus* in the eastern Atlantic (Carlin et al., 2003) and the proposal of its formal taxonomic recognition in the present work. Therefore, the integration of genetic data (capable of identifying cryptic lineages) and traditional taxonomic data is indispensable for a refined knowledge of biodiversity.

The Gulf of Guinea is recognized as an area of utmost importance for biodiversity along the African coast (Briggs, 1974). This area holds the status of a marine biodiversity hotspot due to its high levels of endemism and the threats it faces, making it a priority area for conservation efforts (Roberts et al., 2002). However, despite its significance, this region remains one of the least understood tropical reef environments globally and, unfortunately, holds the second position in the global ranking of marine hotspots most threatened by anthropogenic impacts (Maia et al., 2018). Since 2007, 77 new fish records have been documented in the region, including species at the larger end of the size spectrum, such as the whale shark, *Rhincodon typus* Smith 1828, and the blue marlin, *Makaira nigricans* Lacepède 1802 (Costa et al., 2022; Vasco‐Rodrigues et al., 2016). In recent years, several species from different families were described for the Tropical eastern Atlantic, including gobies, wrasses, jacks, blennies, basses and clingfishes (Fricke, 2007; Smith‐Vaniz & Carpenter, 2007; Kovacic & Schliewen, 2008; Rocha, Brito & Robertson, 2012; Wirtz, 2014; Fricke & Wirtz, 2017, 2023). Other studies have also demonstrated evidence of possible new species that have yet to be taxonomically recognized (e.g. *Ophioblennius* sp., *Scartella* sp., *Hypleurochilus* sp.; Muss et al., 2001; Araujo et al., 2020; Carter et al., 2023). These data indicate that there are still immense gaps in basic faunal inventories regarding the ichthyofauna of the Gulf of Guinea, which desperately needs further studies (Floeter et al., 2008).

## AUTHOR CONTRIBUTIONS

This manuscript was written with contributions from all authors. All authors approved the final version of the manuscript. All authors contributed to the design of the experiments. GSA and CLSS collected morphometric data. GSA performed analysis of the molecular data.

## FUNDING INFORMATION

Fish survey funding for São Tomé was provided by the National Geographic Society under the leadership of Sergio R. Floeter (Grant #7937–05). Financial support to Gabriel S. Araujo was provided by Conselho Nacional de Desenvolvimento Científico e Tecnológico (CNPq) (grant PROTAX #443302/2020), by the Lakeside Foundation (grant #4‐22‐0314) and the São Paulo Research Foundation (FAPESP) (grant #2023/12231–9). Claudio L.S. Sampaio is granted by Fundação de Amparo à Pesquisa do Estado de Alagoas (FAPEAL). Luiz A. Rocha is funded by the California Academy of Sciences Hope For Reefs Initiative. Carlos E.L. Ferreira is continuously granted by CNPq and Fundação Carlos Chagas Filho de Amparo à Pesquisa do Estado do Rio de Janeiro (FAPERJ). The authors acknowledge The Nippon Foundation‐Nekton Ocean Census Programme (https://oceancensus.org/) for supporting the description of this species. This is Ocean Census Species Number 185.

## Supporting information


**TABLE S1.** Species and GenBank/BOLD accession numbers, locality and additional comments regarding the sequences.
