# Supplementary material for: Integrative taxonomy reveals a new species of the soapfish genus Rypticus (Teleostei: Grammistidae) from the eastern Atlantic Ocean
Source: J Fish Biol. 2025 Jul 21;107(5):1543–52. doi: 10.1111/jfb.70132 (PMC12710812; doi:10.1111/jfb.70132)
Supplement: Supplementary file 1 — TABLE S1. Species and GenBank/BOLD accession numbers, locality and additional comments regarding the sequences. [file JFB-107-1543-s001.docx]

Table S*1*. Species and GenBank/BOLD accession numbers, locality, and additional comments regarding the sequences.

| Species | Genbank/BOLD ID | Country | Comments |
| --- | --- | --- | --- |
| *Rypticus africanus* | PV137738 | São Tome and Principe |  |
| *Rypticus africanus* | PV137742 | São Tome and Principe |  |
| *Rypticus africanus* | PV137740 | São Tome and Principe |  |
| *Rypticus africanus* | PV137741 | Cabo Verde | USNM405085, genseq-3 cytochrome oxidase I |
| *Rypticus africanus* | PV137739 | Cabo Verde | USNM405086, genseq-3 cytochrome oxidase I |
| *Rypticus africanus* | JX093905 | Ghana | Paratype MNHN2002-158, genseq-2 cytochrome oxidase I, misidentified as *R. saponaceus* in BOLD |
| *Rypticus bicolor* | LIDMA1263-12 | Ecuador |  |
| *Rypticus bicolor* | LIDMA915-11 | Ecuador |  |
| *Rypticus bicolor* | RDFCA154-05 | Costa Rica |  |
| *Rypticus bicolor* | RDFCA352-05 | Costa Rica |  |
| *Rypticus bicolor* | RDFCA353-05 | Costa Rica |  |
| *Rypticus bicolor* | RDFCA354-05 | Costa Rica |  |
| *Rypticus bistrispinus* | MFLIV246-09 | Mexico |  |
| *Rypticus bistrispinus* | MFLS191-11 | Mexico |  |
| *Rypticus bistrispinus* | BZLWC441-06 | Belize | Identified as *Rypticus* sp. in BOLD |
| *Rypticus bistrispinus* | BZLWD577-08 | Belize | Identified as *Rypticus* sp. in BOLD |
| *Rypticus bornoi* | LIDM1391-08 | Panama |  |
| *Rypticus bornoi* | BZLWC206-06 | Belize |  |
| *Rypticus carpenteri* | BZLWE047-08 | Belize |  |
| *Rypticus carpenteri* | BZLWE230-08 | Belize |  |
| *Rypticus carpenteri* | BZLWE338-08 | Belize |  |
| *Rypticus carpenteri* | CURA302-09 | Curacao |  |
| *Rypticus carpenteri* | CURA303-09 | Curacao |  |
| *Rypticus carpenteri* | LIDM1246-08 | Panama |  |
| *Rypticus carpenteri* | LIDM855-07 | Panama |  |
| *Rypticus carpenteri* | MFLIV109-09 | Mexico |  |
| *Rypticus carpenteri* | SABA002-11 | Netherlands |  |
| *Rypticus carpenteri* | TOBA102-09 | Trinidad and Tobago |  |
| *Rypticus carpenteri* | TOBA103-09 | Trinidad and Tobago |  |
| *Rypticus carpenteri* | TOBA177-09 | Trinidad and Tobago |  |
| *Rypticus carpenteri* | TOBA178-09 | Trinidad and Tobago |  |
| *Rypticus carpenteri* | TOBA198-09 | Trinidad and Tobago |  |
| *Rypticus carpenteri* | UKFBI263-08 | Belize |  |
| *Rypticus carpenteri* | BZLWB140-06 | Belize |  |
| *Rypticus carpenteri* | BZLWE013-08 | Belize |  |
| *Rypticus carpenteri* | CURA301-09 | Curacao |  |
| *Rypticus carpenteri* | CURA304-09 | Curacao |  |
| *Rypticus carpenteri* | TOBA197-09 | Trinidad and Tobago |  |
| *Rypticus carpenter* | UKFBK227-08 | United States | Misidentified as *R. subbifrenatus* in BOLD |
| *Rypticus maculatus* | ANGBF40397-19 | United States | Misidentified as *R. saponaceus* in BOLD |
| *Rypticus maculatus* | CFSAN064-11 | United States | Misidentified as *R. saponaceus* in BOLD |
| *Rypticus maculatus* | FLBAR1278-19 | United States |  |
| *Rypticus maculatus* | FLBAR1779-20 | United States |  |
| *Rypticus nigripinnis* | BMAR2884-22 | Costa Rica |  |
| *Rypticus nigripinnis* | BMAR2885-22 | Costa Rica |  |
| *Rypticus nigripinnis* | BMAR2886-22 | Costa Rica |  |
| *Rypticus nigripinnis* | BMAR2907-22 | Costa Rica |  |
| *Rypticus nigripinnis* | BMAR3078-22 | Costa Rica |  |
| *Rypticus nigripinnis* | BMAR3079-22 | Costa Rica |  |
| *Rypticus randalli* | BAHIA126-14 | Brazil |  |
| *Rypticus randalli* | BAHIA127-14 | Brazil |  |
| *Rypticus randalli* | BAHIA128-14 | Brazil |  |
| *Rypticus randalli* | BAHIA129-14 | Brazil |  |
| *Rypticus randalli* | BAHIA130-14 | Brazil |  |
| *Rypticus randalli* | BAHIA131-14 | Brazil |  |
| *Rypticus randalli* | BAHIA132-14 | Brazil |  |
| *Rypticus randalli* | BAHIA133-14 | Brazil |  |
| *Rypticus randalli* | UKFBI784-08 | Belize | Identified as *R. saponaceus* in BOLD |
| *Rypticus saponaceus* | BAHB215-15 | Bahamas |  |
| *Rypticus saponaceus* | BZLWC138-06 | Belize |  |
| *Rypticus saponaceus* | BZLWE308-08 | Belize |  |
| *Rypticus saponaceus* | CURA013-09 | Curacao |  |
| *Rypticus saponaceus* | CURA104-09 | Curacao |  |
| *Rypticus saponaceus* | SABA093-11 | Netherlands |  |
| *Rypticus saponaceus* | BAHB143-15 | Bahamas |  |
| *Rypticus saponaceus* | BAHB216-15 | Bahamas |  |
| *Rypticus saponaceus* | LIDM1272-08 | Panama |  |
| *Rypticus saponaceus* | LIDM1310-08 | Barbados |  |
| *Rypticus saponaceus* | BAHB237-15 | Bahamas | Indentified as *Rypticus* sp. in BOLD |
| *Rypticus saponaceus* | BAHB279-15 | Bahamas | Indentified as *Rypticus* sp. in BOLD |
| *Rypticus subbifrenatus* | BZLWB212-06 | Belize |  |
| *Rypticus subbifrenatus* | BZLWD190-07 | Belize |  |
| *Rypticus subbifrenatus* | BZLWD191-07 | Belize |  |
| *Rypticus subbifrenatus* | BZLWD192-07 | Belize |  |
| *Rypticus subbifrenatus* | BZLWD200-07 | Belize |  |
| *Rypticus subbifrenatus* | BZLWE059-08 | Belize |  |
| *Rypticus subbifrenatus* | BZLWE082-08 | Belize |  |
| *Rypticus subbifrenatus* | BZLWE373-08 | Belize |  |
| *Rypticus subbifrenatus* | CURA172-09 | Curacao |  |
| *Rypticus subbifrenatus* | CURA173-09 | Curacao |  |
| *Rypticus subbifrenatus* | LIDM021-06 | Panama |  |
| *Rypticus subbifrenatus* | MLIII216-08 | Mexico |  |
| *Rypticus subbifrenatus* | TOBA001-09 | Trinidad and Tobago |  |
| *Rypticus subbifrenatus* | TOBA104-09 | Trinidad and Tobago |  |
| *Rypticus subbifrenatus* | TOBA105-09 | Trinidad and Tobago |  |
| *Rypticus subbifrenatus* | TOBA106-09 | Trinidad and Tobago |  |
| *Rypticus subbifrenatus* | TOBA253-09 | Trinidad and Tobago |  |
| *Rypticus subbifrenatus* | TOBA254-09 | Trinidad and Tobago |  |
| *Rypticus subbifrenatus* | TOBA255-09 | Trinidad and Tobago |  |
| *Rypticus subbifrenatus* | TOBA256-09 | Trinidad and Tobago |  |
| *Rypticus subbifrenatus* | TOBA329-09 | Trinidad and Tobago |  |
| *Rypticus subbifrenatus* | UKFBK020-08 | Belize |  |
| *Rypticus subbifrenatus* | BZLWD247-07 | Belize |  |
| *Rypticus subbifrenatus* | BZLWE012-08 | Belize |  |
| *Rypticus subbifrenatus* | BZLWE058-08 | Belize |  |
| *Rypticus subbifrenatus* | CURA174-09 | Curacao |  |
| *Rypticus subbifrenatus* | CURA175-09 | Curacao |  |
| *Rypticus subbifrenatus* | LIDM1273-08 | Panama |  |
| *Rypticus subbifrenatus* | LIDM1274-08 | Panama |  |
| *Rypticus subbifrenatus* | SMSA397-09 | United States |  |
| *Rypticus subbifrenatus* | TOBA330-09 | Trinidad and Tobago |  |
| *Rypticus subbifrenatus* | TOBA331-09 | Trinidad and Tobago |  |
